# Cerebral Microbleeds in Critically Ill Patients with Respiratory Failure or Sepsis: A Scoping Review

**DOI:** 10.1007/s12028-024-01961-z

**Published:** 2024-03-20

**Authors:** Bing Yu Chen, Johnny Dang, Sung-Min Cho, Mary Pat Harnegie, Ken Uchino

**Affiliations:** 1 Cerebrovascular Center, Neurological Institute, Cleveland Clinic, 9500 Euclid Avenue, Cleveland, OH 44195 USA; 2 Neurological Institute, Cleveland Clinic, Cleveland, OH USA; 3 Divisions of Neurocritical Care and Cardiac Surgery, Departments of Neurology, Surgery, Anesthesia and Critical Care Medicine, Johns Hopkins University, Baltimore, MD USA; 4 Floyd D. Loop Alumni Library, Cleveland Clinic, Cleveland, OH USA

**Keywords:** Critical illness, Respiratory insufficiency, Respiratory distress syndrome, Sepsis, Cerebral hemorrhage

## Abstract

**Background:**

Cerebral microbleeds (CMBs) have been described in critically ill patients with respiratory failure, acute respiratory distress syndrome (ARDS), or sepsis. This scoping review aimed to systematically summarize existing literature on critical illness–associated CMBs.

**Methods:**

Studies reporting on adults admitted to the intensive care unit for respiratory failure, ARDS, or sepsis with evidence of CMBs on magnetic resonance imaging were included for review following a systematic search across five databases (MEDLINE, Embase, Cochrane Central Register of Controlled Trials (CENTRAL), Scopus, and Web of Science) and a two-stage screening process. Studies were excluded if patients’ CMBs were clearly explained by another process of neurological injury.

**Results:**

Forty-eight studies reporting on 216 critically ill patients (mean age 57.9, 18.4% female) with CMBs were included. Of 216, 197 (91.2%) patients developed respiratory failure or ARDS, five (2.3%) patients developed sepsis, and 14 (6.5%) patients developed both respiratory failure and sepsis. Of 211 patients with respiratory failure, 160 (75.8%) patients had coronavirus disease 2019. The prevalence of CMBs among critically ill patients with respiratory failure or ARDS was 30.0% (111 of 370 patients in cohort studies). The corpus callosum and juxtacortical area were the most frequently involved sites for CMBs (64.8% and 41.7% of all 216 patients, respectively). Functional outcomes were only reported in 48 patients, among whom 31 (64.6%) were independent at discharge, four (8.3%) were dependent at discharge, and 13 (27.1%) did not survive until discharge. Cognitive outcomes were only reported in 11 of 216 patients (5.1%), all of whom showed cognitive deficits (nine patients with executive dysfunction and two patients with memory deficits).

**Conclusions:**

Cerebral microbleeds are commonly reported in patients with critical illness due to respiratory failure, ARDS, or sepsis. CMBs had a predilection for the corpus callosum and juxtacortical area, which may be specific to critical illness–associated CMBs. Functional and cognitive outcomes of these lesions are largely unknown.

**Supplementary Information:**

The online version contains supplementary material available at 10.1007/s12028-024-01961-z.

## Introduction

Cerebral microbleeds (CMBs) are accumulations of small hemosiderin deposits correlating with hypointense foci on susceptibility-weighted magnetic resonance imaging (MRI) [1]. CMBs are associated with several medical conditions, including cerebral amyloid angiopathy [2], traumatic brain injury (TBI) [3], and chronic hypertension [4]. CMBs have been reported in critically ill patients among whom an etiologic explanation for cerebral microvascular injuries was not found [5, 6]. Common factors in these patient populations included the presence of respiratory failure or sepsis, possibly causing hypoxemia-induced microangiopathy or sepsis-related coagulopathy [6]. The objective of this study was to conduct a scoping review describing available evidence on CMBs in critically ill patients with respiratory failure, including acute respiratory distress syndrome (ARDS) or sepsis.

## Methods

### Design

This scoping review was conducted in accordance with recommendations from the Joanna Briggs Institute [7] and the Preferred Reporting Items for Systematic Reviews and Meta-Analyses extension for scoping reviews [8] (Supplemental Table S1). As per these guidelines, a formal bias appraisal was not necessary and was not completed. Institutional review board approval was not required for conducting this review. Inclusion criteria were studies with patients aged 18 or older; who were critically ill due to respiratory failure, including ARDS or sepsis, requiring admission to the intensive care unit (ICU); and who showed evidence of CMBs on MRI. Studies were excluded if patients’ CMBs were clearly explained by another process of neurological injury, such as acute cerebral infarction, TBI, encephalitis, anoxic cerebral injury due to cardiac arrest, and infective endocarditis. All study designs were included except for reviews, guidelines, and commentaries. Abstracts were included if results were not also published as articles. Included studies must be published in English.

### Search Strategy and Selection

Five databases, MEDLINE (1946 to present), Embase (1974 to present), Cochrane Central Register of Controlled Trials (CENTRAL) (1996 to present), Scopus (2000 to present), and Web of Science (1975 to present), were searched from their dates of inception until June 2, 2023. Key concepts related to microbleeds, critical illness, respiratory failure, ARDS, and sepsis were mapped to keywords and Medical Subject Headings terms. This search strategy was developed in collaboration with a professional librarian (MPH) specialized in systemic reviews of medical literature. An example of search strategy used in MEDLINE is provided in Supplemental Table S2. Using a two-stage screening process, two co-authors (BYC and JD) independently selected relevant studies for subsequent data charting from a list of all studies identified using search strategies. In the first stage, studies were excluded based on abstract screening. In the second stage, studies were excluded based on full-text review. Screening was conducted using Covidence Systematic Review software. Any disagreement between the two co-authors was resolved by consensus.

### Data Charting and Synthesis

Two co-authors (BYC and JD) independently performed data charting. Final data charting was completed and verified by the first author. Synthesis and comparison of clinical, paraclinical, radiological, and prognostic data were completed using representative tables and figures. CMB was defined according to Greenberg’s criteria [9]. ARDS was defined according to the Berlin definition [10]. Disseminated intravascular coagulation was defined according to the International Society on Thrombosis and Haemostasis Criteria [11]. Functional outcomes were trichotomized into independence (modified Rankin Scale [mRS] 0–2), dependence (mRS 3–5), or death at the time of hospital discharge if outcome data from included studies permitted such characterization. Cognitive deficits during postdischarge follow-up assessment, if described in included studies, were classified into executive, memory, visuospatial, language, praxis, or behavioral dysfunction.

## Results

**Fig. 1:**
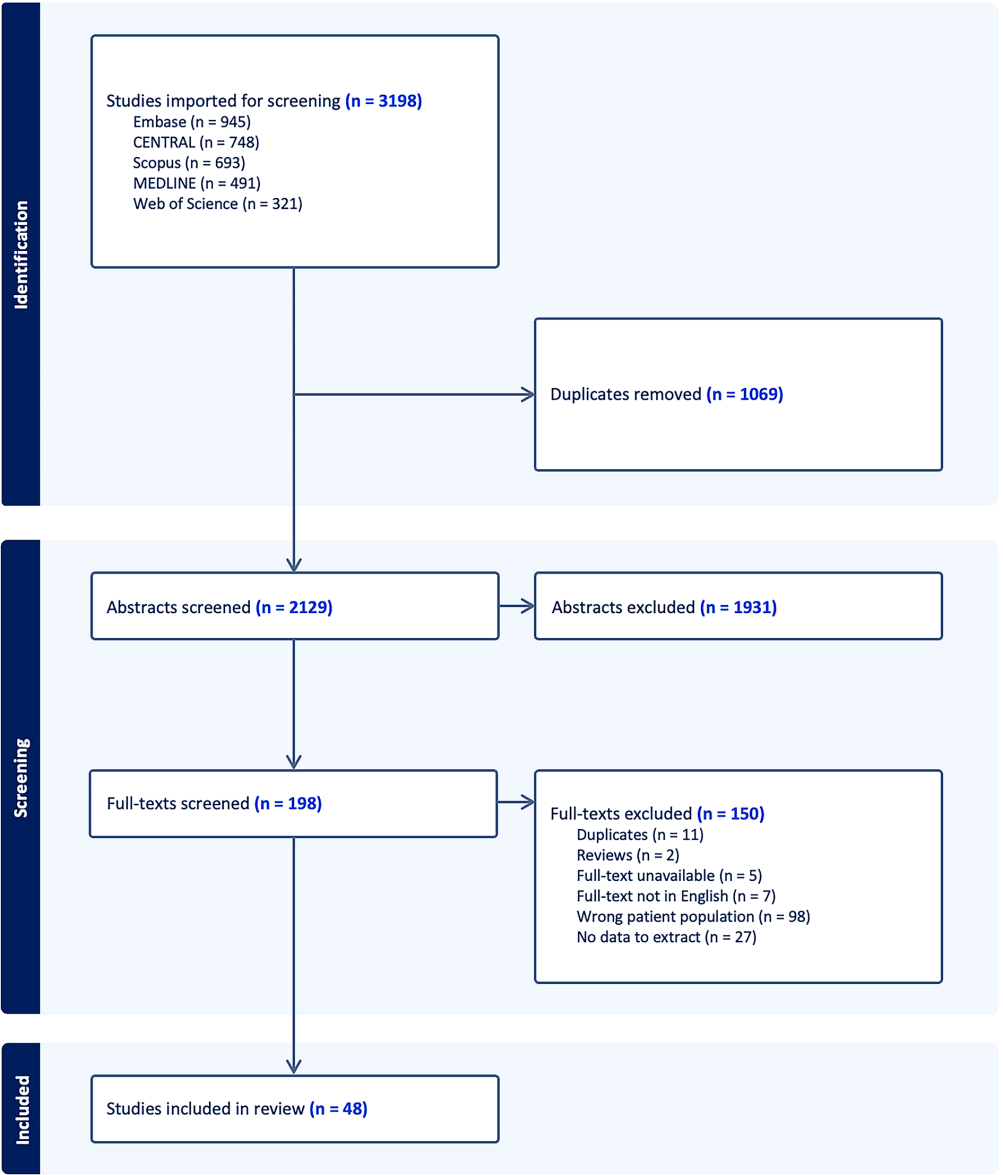
Preferred Reporting Items for Systematic Reviews and Meta-Analyses flowchart of study selection. CENTRAL, Cochrane Central Register of Controlled Trials

In total, 48 studies were included for data charting and synthesis (Fig. 1), comprising 28 case reports (58 patients), two case–control studies (31 patients), 16 retrospective cohort studies (66 patients), and two prospective cohort studies (61 patients), published between 2012 and 2023 from 16 countries. Thirty-one studies described at least one patient diagnosed with coronavirus disease 2019 (COVID-19). A summary of all included studies is listed in Supplemental Table S3.

A descriptive analysis of individual patient data extracted from included studies is shown in Table 1. A total of 216 critically ill patients with respiratory failure, ARDS, or sepsis and findings of CMBs on MRI were included (mean age 57.9, 18.4% female). One hundred ninety-seven (91.2%) patients had developed respiratory failure or ARDS, five (2.3%) patients had developed sepsis, and 14 (6.5%) patients had developed both respiratory failure and sepsis, requiring ICU admission. One hundred sixty (74.1%) patients were diagnosed with COVID-19, and 172 (79.6%) patients were diagnosed with any form of pneumonia. During hospitalization, 114 (52.8%) patients required mechanical ventilation, 49 (22.7%) patients required extracorporeal membrane oxygenation (ECMO), 27 (12.5%) patients underwent dialysis, and 35 (16.2%) patients received therapeutic anticoagulation. Only eight (3.7%) patients met diagnostic criteria for disseminated intravascular coagulation.

**Table 1:** Patient characteristics, course of hospitalization, cerebral microbleeds location, and outcomes

Characteristic	All (*N* = 216)^a^	COVID-19 (*n* = 160)^a^	Non-COVID-19 (*n* = 56)^a^
Demographics			
Age, mean (SD) (year)	57.9 (11.9), *n* = 137	61.7 (8.9), *n* = 92	50.1 (14.1), *n* = 45
Female sex, *n* (%)	23 (18.4), *n* = 125	16 (17.2), *n* = 93	7 (21.9), *n* = 32
Comorbidities, *n* (%)			
Hypertension	29 (13.4)	26 (16.3)	3 (5.4)
Diabetes mellitus	11 (5.1)	10 (6.3)	1 (1.8)
Dyslipidemia	16 (7.4)	15 (9.4)	1 (1.8)
Atrial fibrillation	1 (0.5)	1 (0.6)	0 (0.0)
Coronary artery disease	5 (2.3)	4 (2.5)	1 (1.8)
Hematological disease	14 (6.5)	3 (1.9)	11 (19.6)
Hypothyroidism	6 (2.8)	5 (3.1)	1 (1.8)
Chronic obstructive pulmonary disease	6 (2.8)	6 (3.8)	0 (0.0)
Liver disease	2 (0.9)	1 (0.6)	1 (1.8)
Kidney disease	3 (1.4)	3 (1.9)	0 (0.0)
Reason for ICU admission, *n* (%)			
Respiratory failure or ARDS alone	197 (91.2)	155 (96.9)	42 (75.0)
Sepsis alone	5 (2.3)	0 (0.0)	5 (8.9)
Both respiratory failure and sepsis	14 (6.5)	5 (3.1)	9 (16.1)
Course of hospitalization, *n* (%)			
Pneumonia	172 (79.6)	156 (97.5)	16 (28.6)
COVID-19	160 (74.1)	160 (100.0)	0 (0.0)
Mechanical ventilation	114 (52.8)	66 (41.3)	48 (85.7)
Extracorporeal membrane oxygenation	49 (22.7)	11 (6.9)	38 (67.9)
Dialysis	27 (12.5)	25 (15.6)	2 (3.6)
Anticoagulation	35 (16.2)	35 (21.9)	0 (0.0)
Disseminated intravascular coagulopathy	8 (3.7)	1 (0.6)	7 (12.5)
Neuroimaging, *n* (%)			
In-hospital MRI	154 (71.3)	98 (61.3)	56 (100.0)
Outpatient MRI	62 (28.7)	62 (38.7)	0 (0.0)
Microbleed location reported			
Cortex	31 (14.4)	20 (12.5)	11 (19.6)
Juxtacortical area	90 (41.7)	66 (41.3)	24 (42.9)
Periventricular area	12 (5.6)	11 (6.9)	1 (1.8)
Basal ganglia, internal capsules, and thalami	80 (37.0)	65 (40.6)	15 (26.8)
Corpus callosum	140 (64.8)	105 (65.6)	35 (62.5)
Brainstem	46 (21.3)	38 (23.8)	8 (14.3)
Cerebellum and cerebellar peduncles	67 (31.0)	50 (31.3)	17 (30.4)
Outcomes			
Length of ICU stay, mean (SD) (d)	27.8 (8.1), *n* = 48	27.9 (9.4), *n* = 35	27.5 (1.9), *n* = 13
Length of hospitalization, mean (SD) (d)	42.9 (15.5), *n* = 33	44.4 (14.4), *n* = 31	19.0 (15.6), *n* = 2
Modified Rankin Scale 0–2, *n* (%)	31 (64.6), *n* = 48	21 (67.7), *n* = 31	10 (58.8), *n* = 17
Modified Rankin Scale 3–5, *n* (%)	4 (8.3), *n* = 48	2 (6.5), *n* = 31	2 (11.8), *n* = 17
Death, *n* (%)	13 (27.1), *n* = 48	8 (25.8), *n* = 31	5 (29.4), *n* = 17
Executive dysfunction, *n* (%)	9 (81.8), *n* = 11	3 (75.0), *n* = 4	6 (85.7), *n* = 7
Memory dysfunction, *n* (%)	2 (18.2), *n* = 11	1 (25.0), *n* = 4	1 (14.3), *n* = 7

ARDS, acute respiratory distress syndrome; CMB, cerebral microbleed; COVID-19, coronavirus disease 2019; ICU, intensive care unit; MRI, magnetic resonance imaging; SD, standard deviation

^a^Denominators for all COVID-19 and non-COVID-19 populations were *n* = 216, *n* = 160, and *n* = 56, respectively, unless otherwise specified

Of seven studies providing data for estimating the prevalence of CMBs among critically ill patients with respiratory failure or ARDS undergoing MRI, the prevalence was 30.0% (111 of 370 patients in cohort studies, range 3.3–60.6%) [12–18]. The prevalence of CMBs among patients with COVID-19 was 32.0% (99 of 309 patients, range 3.3–60.6%), and the prevalence among patients without COVID-19 was 19.7% (12 of 61 patients, only one study reported prevalence).

**Fig. 2:**
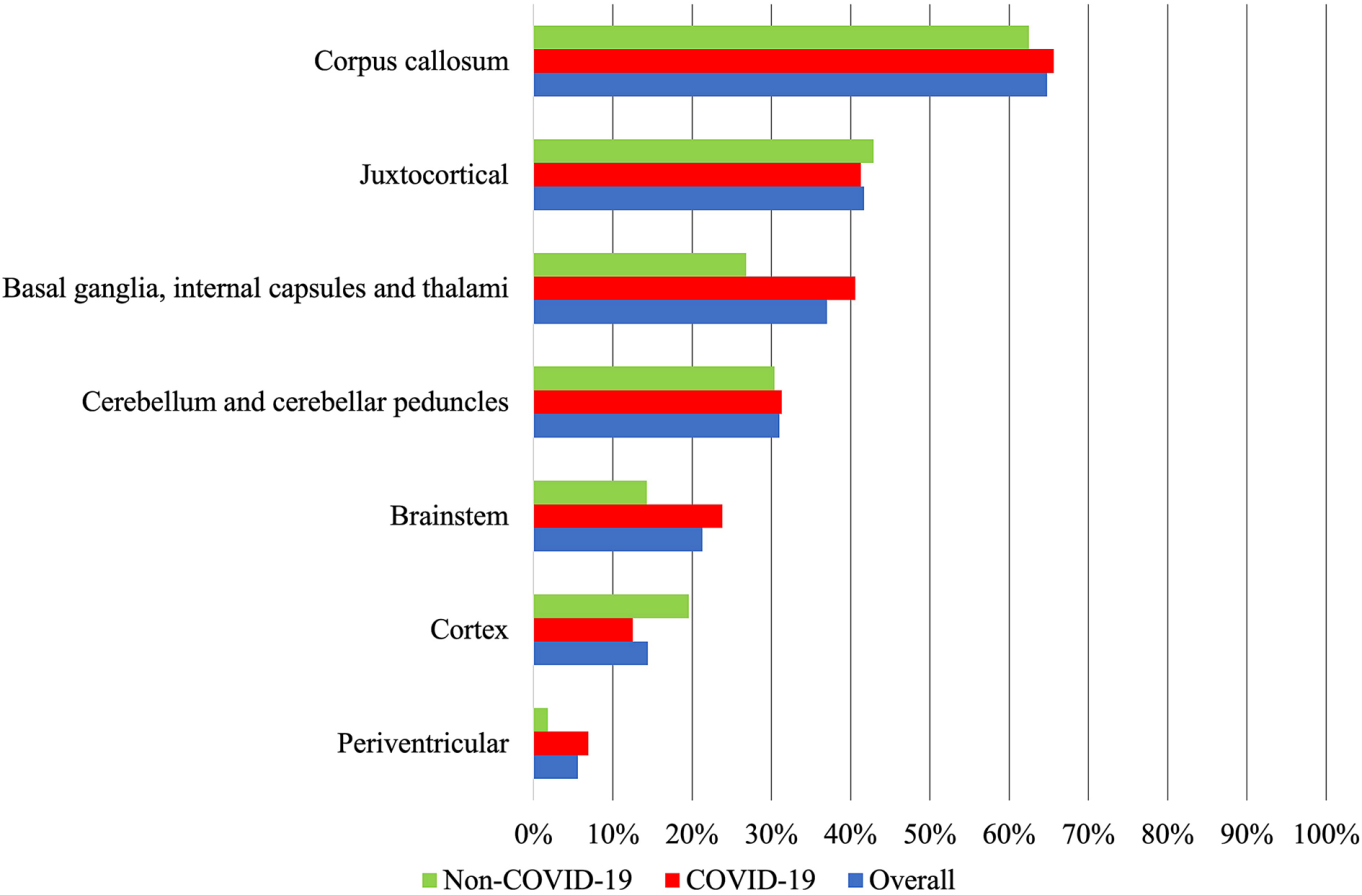
Cerebral microbleeds location. COVID-19, coronavirus disease 2019

One hundred fifty-four (71.3%) patients had at least one MRI scan of the head during hospitalization (median time 15 days after beginning of hospitalization, range 0–76 days, timing reported in 21 patients), whereas the remaining 62 (28.7%) patients only underwent MRI scan following discharge (median time 5 months after discharge, range 1–36 months, timing reported in 34 patients). The corpus callosum (140 of 216 patients, 64.8%) and juxtacortical area (90 of 216 patients, 41.7%) were the most frequently involved sites for CMBs (Fig. 2). CMBs were often described as diffuse and innumerable by most studies, and thus the number of CMBs in each location was not quantified in this study. In three studies, MRI scans were performed both in-hospital and after hospital discharge, and no change in the number and locations of CMBs was observed in all 19 patients [6, 19, 20]. Regardless of whether patients were diagnosed with COVID-19, the most common sites of involvement for CMBs were the corpus callosum (105 of 160 [65.6%] patients with COVID-19 and 35 of 56 [62.5%] patients without COVID-19) and juxtacortical area (66 of 160 [41.3%] patients with COVID-19 and 24 of 56 [42.9%] patients without COVID-19).

Functional outcomes were only reported in 48 patients (22.2% of all patients with CMBs), among whom 31 (64.6%) were independent at discharge (mRS 0–2), four (8.3%) were dependent at discharge (mRS 3–5), and 13 (27.1%) did not survive until discharge. In the only study comparing outcomes between patients with and without CMBs, mortality at discharge was not different between the two groups (41.7% and 34.7%, respectively, *p* = 0.652) [18]. Cognitive assessment was reported in 11 patients (5.1% of all patients with CMBs) during follow-up (timing unavailable). All 11 patients had detectable cognitive deficits, nine of whom had executive deficits and two of whom had memory deficits. No prehospitalization cognitive assessment was available for all 11 patients. No study comparing cognitive outcomes between patients with and without CMBs was found.

## Discussion

Our scoping review of CMBs on MRI among patients with critical illness due to respiratory failure, ARDS, or sepsis resulted in 216 patients from 48 studies, composed of 160 patients with COVID-19 and 56 patients without COVID-19 infection. It is possible that patients with COVID-19 were overrepresented in this review, given the recent COVID-19 pandemic. We found that the corpus callosum and the juxtacortical area were the most common sites of involvement by CMBs. The prevalence and common sites of location and the CMB do not appear to be different among the subgroup analysis of COVID-19 and non-COVID-19 cases, suggesting CMBs are likely reflective of respiratory failure, rather than COVID-19 infection.

The corpus callosum was the most common site, found in 64.8% of all patients, 65.6% of patients with COVID-19, and 62.5% of patients without COVID-19. Callosal involvement by CMBs is commonly associated with TBI and generally not associated with other causes of CMBs (e.g., cerebral amyloid angiopathy, chronic hypertension, infective endocarditis, radiation) [21]. Patients with TBI were excluded from this review. CMBs in the corpus callosum have been described in patients with high-altitude cerebral edema [22]. It has been hypothesized that severe hypoxemia in both patients with high-altitude cerebral edema and critically ill patients may cause cerebral capillary vasodilation, disruption of the blood–brain barrier due to hydrostatic forces or chemical effects from hypoxia, and ultimately extravasation of erythrocytes, particularly at the corpus callosum [6]. In patients with anoxic brain injury, which represents a severe form of cerebral hypoxia, CMBs had special predilection for the splenium of corpus callosum, thus supporting the hypoxemia hypothesis [23]. Other hypothetical mechanisms for the presence of CMBs include disseminated intravascular coagulation, platelet dysfunction, and COVID-19-related endothelial inflammation [24]. Iatrogenic causes of CMBs, such as ventilation-induced cerebral venous hypertension [5], ECMO [25], or dialysis [26], have also been reported. In summary, hypoxemia and sepsis may result in disruption of the blood–brain barrier, coagulopathy, endothelial dysfunction, and CMBs.

Regardless of the pathophysiology, critical illness–associated CMB could be considered a distinct entity given its unique preferential involvement of the corpus callosum and persistent CMB location pattern among both patients with COVID-19 and patients without COVID-19. However, because only 2.3% of patients had isolated sepsis without respiratory failure or ARDS, it is difficult to establish whether patients with sepsis share a similar pattern of CMB location as patients with respiratory failure. Furthermore, the low number of published cases describing CMBs in patients with isolated sepsis may raise doubt on whether severe sepsis is strongly associated with CMBs. Critical illness–associated CMB may be present mainly in respiratory failure or ARDS. This review only included patients with respiratory failure, ARDS, or sepsis, mirroring a similar patient population described in the case series by Fanou et al. [6] and review by Puy et al. [21]. It is unknown whether other causes of critical illness, such as cardiogenic shock, are associated with CMBs.

CMBs have been associated with worse cognitive function in other contexts, but little is known about the clinical significance of the presence of CMBs among patients in the ICU [27]. Only 48 of 216 patients had any description of functional outcomes following discharge. Cognitive deficits were reported in only 5.1% of patients, which likely were underestimated because of underreporting and lack of follow-up. No comparative study of cognitive outcomes between critically ill patients with and without CMBs was found in our systematic review. Given the paucity of data, no conclusion regarding cognitive and functional outcomes may be drawn from this review.

Our article has several limitations and thus should only be regarded as hypothesis generating. Most of the included studies were case reports, with potential reporting bias, but the majority of patients were from prospective or retrospective cohort studies. The overall sample size (*N* = 216) was small, and thus any finding should be interpreted with caution. This was representative of the current literature on this important topic and that was why we performed a scoping review and not a systematic review or meta-analysis. There was methodological heterogeneity across studies. For example, 28.7% of included patients did not have neuroimaging during hospitalization but only after discharge. Because CMB distribution may evolve over time following acute critical illness, this heterogeneity in timing of neuroimaging may introduce bias. Of note, 19 patients pooled from three studies did not demonstrate any difference in CMB patterns between in-hospital and follow-up neuroimaging [6, 19, 20]. The prevalence of associated patient characteristics, course of hospitalization, neuroimaging, and outcomes may be underestimated because some studies might not have reported the variable. For example, the prevalence of mechanical ventilation of 52.8% among critically ill patients with respiratory failure or sepsis is low. Our study included patients who may have other clinical characteristics or treatments known to be associated with CMBs, such as sickle cell anemia [28] and ECMO [25], thus representing potential confounding factors. Well-designed prospective registries of critically ill patients with serial inpatient and outpatient neuroimaging with susceptibility sequences and cognitive and functional outcomes assessment during follow-up are needed to inform on the true prevalence of CMBs in this patient population and the clinical and functional significance of CMBs so that timely rehabilitation and appropriate services on discharge may be provided. This may be particularly important in nonhypoxic critically ill patients, such as patients with sepsis. The available retrospective data were even more sparse for these populations, as they may have been less likely to undergo neuroimaging than their hypoxic counterparts.

## Conclusions

In conclusion, a subset of critically ill patients with respiratory failure harbor CMBs with predilection for the corpus callosum and juxtacortical area in the brain. The true prevalence, clinical correlates, and prognostic significance of these CMBs are important gaps in knowledge that need to be addressed in the future.

## Supplementary Information

Below is the link to the electronic supplementary material.

Supplementary file1 (DOCX 87 kb)
