# Supplementary material for: Cerebral Microbleeds in Critically Ill Patients with Respiratory Failure or Sepsis: A Scoping Review
Source: Neurocrit Care. 2024 Mar 20;41(2):533–40. doi: 10.1007/s12028-024-01961-z (PMC11377596; doi:10.1007/s12028-024-01961-z)
Supplement: Supplementary file 1 — Supplementary file1 (DOCX 87 kb) [file 12028_2024_1961_MOESM1_ESM.docx]

Supplemental Table S1. Preferred Reporting Items for Systematic reviews and Meta-Analyses extension for Scoping Reviews (PRISMA-ScR) Checklist^1^

| **SECTION** | **ITEM** | **PRISMA-ScR CHECKLIST ITEM** | **REPORTED ON PAGE #** |
| --- | --- | --- | --- |
| **TITLE** | | | |
| Title | 1 | Identify the report as a scoping review. | 1 |
| **ABSTRACT** | | | |
| Structured summary | 2 | Provide a structured summary that includes (as applicable): background, objectives, eligibility criteria, sources of evidence, charting methods, results, and conclusions that relate to the review questions and objectives. | 3-4 |
| **INTRODUCTION** | | | |
| Rationale | 3 | Describe the rationale for the review in the context of what is already known. Explain why the review questions/objectives lend themselves to a scoping review approach. | 5 |
| Objectives | 4 | Provide an explicit statement of the questions and objectives being addressed with reference to their key elements (e.g., population or participants, concepts, and context) or other relevant key elements used to conceptualize the review questions and/or objectives. | 5 |
| **METHODS** | | | |
| Protocol and registration | 5 | Indicate whether a review protocol exists; state if and where it can be accessed (e.g., a Web address); and if available, provide registration information, including the registration number. | Not reported |
| Eligibility criteria | 6 | Specify characteristics of the sources of evidence used as eligibility criteria (e.g., years considered, language, and publication status), and provide a rationale. | 5-6 |
| Information sources* | 7 | Describe all information sources in the search (e.g., databases with dates of coverage and contact with authors to identify additional sources), as well as the date the most recent search was executed. | 6 |
| Search | 8 | Present the full electronic search strategy for at least 1 database, including any limits used, such that it could be repeated. | Table S2 |
| Selection of sources of evidence† | 9 | State the process for selecting sources of evidence (i.e., screening and eligibility) included in the scoping review. | 6 |
| Data charting process‡ | 10 | Describe the methods of charting data from the included sources of evidence (e.g., calibrated forms or forms that have been tested by the team before their use, and whether data charting was done independently or in duplicate) and any processes for obtaining and confirming data from investigators. | 6-7 |
| Data items | 11 | List and define all variables for which data were sought and any assumptions and simplifications made. | 6-7 |
| Critical appraisal of individual sources of evidence§ | 12 | If done, provide a rationale for conducting a critical appraisal of included sources of evidence; describe the methods used and how this information was used in any data synthesis (if appropriate). | Not reported |
| Synthesis of results | 13 | Describe the methods of handling and summarizing the data that were charted. | 6-7 |
| **RESULTS** | | | |
| Selection of sources of evidence | 14 | Give numbers of sources of evidence screened, assessed for eligibility, and included in the review, with reasons for exclusions at each stage, ideally using a flow diagram. | Figure 1 |
| Characteristics of sources of evidence | 15 | For each source of evidence, present characteristics for which data were charted and provide the citations. | Table S3 |
| Critical appraisal within sources of evidence | 16 | If done, present data on critical appraisal of included sources of evidence (see item 12). | Not reported |
| Results of individual sources of evidence | 17 | For each included source of evidence, present the relevant data that were charted that relate to the review questions and objectives. | Table S3 |
| Synthesis of results | 18 | Summarize and/or present the charting results as they relate to the review questions and objectives. | 7-9; Table 1; Figure 2 |
| **DISCUSSION** | | | |
| Summary of evidence | 19 | Summarize the main results (including an overview of concepts, themes, and types of evidence available), link to the review questions and objectives, and consider the relevance to key groups. | 9-11 |
| Limitations | 20 | Discuss the limitations of the scoping review process. | 11 |
| Conclusions | 21 | Provide a general interpretation of the results with respect to the review questions and objectives, as well as potential implications and/or next steps. | 11 |
| **FUNDING** | | | |
| Funding | 22 | Describe sources of funding for the included sources of evidence, as well as sources of funding for the scoping review. Describe the role of the funders of the scoping review. | 12 |

JBI = Joanna Briggs Institute; PRISMA-ScR = Preferred Reporting Items for Systematic reviews and Meta-Analyses extension for Scoping Reviews.

* Where *sources of evidence* (see second footnote) are compiled from, such as bibliographic databases, social media platforms, and Web sites.

† A more inclusive/heterogeneous term used to account for the different types of evidence or data sources (e.g., quantitative and/or qualitative research, expert opinion, and policy documents) that may be eligible in a scoping review as opposed to only studies. This is not to be confused with *information sources* (see first footnote).

‡ The frameworks by Arksey and O’Malley (6) and Levac and colleagues (7) and the JBI guidance (4, 5) refer to the process of data extraction in a scoping review as data charting*.*

§ The process of systematically examining research evidence to assess its validity, results, and relevance before using it to inform a decision. This term is used for items 12 and 19 instead of "risk of bias" (which is more applicable to systematic reviews of interventions) to include and acknowledge the various sources of evidence that may be used in a scoping review (e.g., quantitative and/or qualitative research, expert opinion, and policy document).

Supplemental Table S2. MEDLINE search strategy

| 1 | (microbleed* or micro-bleed* or CMB*).mp. |
| --- | --- |
| 2 | (microhemorrhage* or micro-hemorrhage* or microhaemorrhage* or micro-haemorrhage*).mp. |
| 3 | (SWI or "susceptibility weighted" or "susceptibility-weighted" or GRE or "gradient recalled" or "gradient-recalled" or "gradient echo" or "gradient-echo").mp. |
| 4 | exp Cerebral Small Vessel Diseases/ |
| 5 | or/1-4 |
| 6 | (ICU* or MICU* or MSICU* or "intensive care" or "intensive-care").mp. |
| 7 | exp Intensive Care Units/ |
| 8 | ("critical care" or "critical-care").mp. |
| 9 | exp Critical Care/ |
| 10 | ((critical* adj3 (ill* or condition*)) or "critical*-ill*").mp. |
| 11 | exp Critical Illness/ |
| 12 | or/6-11 |
| 13 | (ARDS* or "respirat* distress* syndrome*").mp. |
| 14 | exp Respiratory Distress Syndrome/ |
| 15 | (((respirat* or breath* or ventilat*) adj3 (fail* or distress* or insufficienc* or decompensat* or depress*)) or (hypercapni* or hypoxemi* or hypoxaemi* or dyspn*)).mp. |
| 16 | exp Respiratory Insufficiency/ |
| 17 | or/13-16 |
| 18 | (sepsis or septic* or bacteremia* or bacteraemia* or pyemia* or pyaemia* or pyohemia* or (blood* adj3 (infect* or poison*))).mp. |
| 19 | exp Sepsis/ |
| 20 | exp Bacteremia/ |
| 21 | or/18-20 |
| 22 | 12 or 17 or 21 |
| 23 | 5 and 22 |

Supplemental Table S3. Characteristics of Included Studies

| **First Author** | **Year** | **Country** | **Study design** | **Number of patients** | **COVID-19** | **Reason for ICU admission** |
| --- | --- | --- | --- | --- | --- | --- |
| Agarwal^2^ | 2020 | USA | Retrospective cohort study | 1 | All | ARDS |
| Aragao^3^ | 2021 | Brazil | Retrospective cohort study | 3* | All | Respiratory failure |
| Backman^4^ | 2022 | Sweden | Case report | 1 | All | ARDS |
| Breit^5^ | 2018 | USA | Case report | 1 | None | ARDS |
| Buttner^6^ | 2021 | Germany | Retrospective cohort study | 4 | All | ARDS |
| Cannac^7^ | 2020 | France | Case report | 1 | All | ARDS |
| Chougar^8^ | 2020 | France | Retrospective cohort study | 4 | All | ARDS |
| Conklin^9^ | 2021 | USA | Retrospective cohort study | 11 | All | ARDS |
| Correa^10^ | 2012 | Brazil | Case report | 1 | None | Sepsis |
| De Stefano^11^ | 2020 | Switzerland | Case report | 1 | All | ARDS |
| Dhillon^12^ | 2020 | United Kingdom | Case report | 1 | All | Respiratory failure |
| Dixon^13^ | 2020 | United Kingdom | Case report | 9 | All | ARDS |
| El Beltagi^14^ | 2021 | Qatar | Case report | 1 | All | ARDS |
| Ermis^15^ | 2021 | Germany | Retrospective cohort study | 2 | All | ARDS |
| Fanou^16^ | 2017 | Canada | Case report | 12 | None | Respiratory failure |
| Fitsiori^17^ | 2020 | Switzerland | Case report | 9 | All | ARDS |
| Gedansky^18^ | 2022 | USA | Retrospective cohort study | 12* | None | ARDS |
| Gijs^19^ | 2018 | Belgium | Case report | 1 | None | ARDS |
| Gupta^20^ | 2020 | USA | Case report | 1 | All | Respiratory failure |
| Hall^21^ | 2018 | USA | Case report | 1 | None | ARDS |
| Haroon^22^ | 2020 | Qatar | Case report | 1 | All | ARDS |
| Ippolito^23^ | 2022 | Germany | Retrospective cohort study | 2* | All | Respiratory failure |
| Jegatheeswaran^24^ | 2022 | Canada | Retrospective cohort study | 4 | All | Respiratory failure |
| Keller^25^ | 2020 | Switzerland | Case report | 1 | All | Respiratory failure |
| Klinkhammer^26^ | 2023 | The Netherlands | Prospective cohort study | 60* | All | Respiratory failure |
| Kurki^27^ | 2021 | Finland | Prospective cohort study | 1 | All | Respiratory failure |
| Le Guennec^28^ | 2015 | France | Case report | 3 | None | Pancreatitis |
| Lersy^29^ | 2021 | France | Case-control study | 19*† | All | ARDS |
| Lersy^30^ | 2022 | France | Retrospective cohort study | 6† | All | ARDS |
| Lin^31^ | 2020 | USA | Retrospective cohort study | 3* | All | ARDS |
| Manelli^32^ | 2021 | Italy | Case report | 1 | All | ARDS, sepsis |
| Myers^33^ | 2021 | United Kingdom | Case report | 1 | All | Respiratory failure |
| Neligan^34^ | 2014 | United Kingdom | Case report | 1 | None | Sepsis |
| Ong^35^ | 2021 | USA | Retrospective cohort study | 1 | None | ARDS |
| Parasram^36^ | 2022 | USA | Case report | 1 | All | ARDS |
| Radmanesh^37^ | 2020 | USA | Retrospective cohort study | 1 | All | ARDS, sepsis |
| Riech^38^ | 2015 | Germany | Retrospective cohort study | 14 | None | ARDS |
| Salam^39^ | 2017 | USA | Case report | 1 | None | Sepsis |
| Shafaat^40^ | 2020 | USA | Case report | 1 | None | Respiratory failure |
| Shah^41^ | 2015 | USA | Case report | 1 | None | Respiratory failure |
| Shindo^42^ | 2018 | Japan | Case report | 1 | None | Sepsis |
| Shoskes^43^ | 2022 | USA | Case-control study | 12* | All | ARDS |
| Sierra-Gomez^44^ | 2022 | Spain | Case report | 1 | None | Sepsis |
| Thurnher^45^ | 2021 | Austria | Retrospective cohort study | 2 | Some | ARDS |
| Toeback^46^ | 2021 | Belgium | Case report | 2 | All | ARDS |
| Topiwala^47^ | 2022 | USA | Retrospective cohort study | 2 | None | ARDS, sepsis |
| Trevino-Peinado^48^ | 2015 | Spain | Case report | 1 | None | Sepsis |
| Vattoth^49^ | 2020 | Qatar | Case report | 1 | All | Respiratory failure, sepsis |

*The prevalence of CMB among critically ill patients undergoing MRI was calculated based on 7 studies (Aragao et al, 3 patients with CMB out of 11 patients undergoing MRI; Gedansky et al, 12 of 61; Ippolito et al, 2 of 5; Klinkhammer et al, 60 of 99; Lersy et al, 19 of 80; Lin et al, 3 of 92; Shoskes et al, 12 of 22).

†The 6 patients from Lersy et al 2022 were also included among the 19 patients from Lersy et al 2021, and were not considered unique patients in the final data analysis.

Abbreviations: ARDS, acute respiratory distress syndrome; CMB, cerebral microbleed; COVID-19, Coronavirus Disease 2019; ICU, intensive care unit; USA, United States of America

Supplemental references

1. Tricco AC, Lillie E, Zarin W, et al. PRISMA Extension for Scoping Reviews (PRISMA-ScR): Checklist and Explanation. *Ann Intern Med*. 2018;169(7):467-473. doi:10.7326/M18-0850

2. Agarwal S, Jain R, Dogra S, et al. Cerebral Microbleeds and Leukoencephalopathy in Critically Ill Patients With COVID-19. *Stroke*. 2020;51(9):2649-2655. doi:10.1161/STROKEAHA.120.030940

3. Aragao M de FVV, Leal M de C, Andrade PHP, et al. Clinical and Radiological Profiles of COVID-19 Patients with Neurological Symptomatology: A Comparative Study. *Viruses*. 2021;13(5). doi:10.3390/v13050845

4. Backman L, Moller MC, Thelin EP, et al. Monthlong Intubated Patient with Life-Threatening COVID-19 and Cerebral Microbleeds Suffers Only Mild Cognitive Sequelae at 8-Month Follow-up: A Case Report. *Arch Clin Neuropsychol Off J Natl Acad Neuropsychol*. 2022;37(2):531-543. doi:10.1093/arclin/acab075

5. Breit H, Jhaveri M, John S. Concomitant delayed posthypoxic leukoencephalopathy and critical illness microbleeds. *Neurol Clin Pract*. 2018;8(5):e31-e33. doi:10.1212/CPJ.0000000000000513

6. Buttner L, Bauknecht HC, Fleckenstein FN, et al. Neuroimaging Findings in Conjunction with Severe COVID-19. *Neuroradiol Befunde Im Zusammenhang Mit Schwerer COVID-19-Erkrank*. 2021;193(7):822-829. doi:10.1055/a-1345-9784

7. Cannac O, Martinez-Almoyna L, Hraiech S. Critical illness-associated cerebral microbleeds in COVID-19 acute respiratory distress syndrome. *Neurology*. 2020;95(11):498-499. doi:10.1212/WNL.0000000000010537

8. Chougar L, Shor N, Weiss N, et al. Retrospective Observational Study of Brain Magnetic Resonance Imaging Findings in Patients with Acute SARS-CoV-2 Infection and Neurological Manifestations. *Radiology*. Published online 2020:202422. doi:10.1148/radiol.2020202422

9. Conklin J, Frosch MP, Mukerji SS, et al. Susceptibility-weighted imaging reveals cerebral microvascular injury in severe COVID-19. *J Neurol Sci*. 2021;421:117308. doi:10.1016/j.jns.2021.117308

10. Correa DG, Cruz Junior LCH, Bahia PRV, Gasparetto EL. Intracerebral microbleeds in sepsis: Susceptibility-weighted mr imaging findings. *Arq Neuropsiquiatr*. 2012;70(11):903-904. doi:10.1590/S0004-282X2012001100017

11. De Stefano P, Nencha U, De Stefano L, Megevand P, Seeck M. Focal EEG changes indicating critical illness associated cerebral microbleeds in a Covid-19 patient. *Clin Neurophysiol Pract*. 2020;5:125-129. doi:10.1016/j.cnp.2020.05.004

12. Dhillon PS, Chattopadhyay A, Dineen RA, Lenthall R. Hemorrhagic neurologic manifestations in covid-19: An isolated or multifactorial cause? *Am J Neuroradiol*. 2020;41(11):E89-E90. doi:10.3174/ajnr.A6795

13. Dixon L, McNamara C, Gaur P, et al. Cerebral microhaemorrhage in COVID-19: A critical illness related phenomenon? *Stroke Vasc Neurol*. 2020;5(4):315-322. doi:10.1136/svn-2020-000652

14. El Beltagi AH, Vattoth S, Abdelhady M, et al. Spectrum of neuroimaging findings in COVID-19. *Br J Radiol*. 2021;94(1117):20200812. doi:10.1259/bjr.20200812

15. Ermis U, Rust MI, Bungenberg J, et al. Neurological symptoms in COVID-19: a cross-sectional monocentric study of hospitalized patients. *Neurol Res Pract*. 2021;3(1):17. doi:10.1186/s42466-021-00116-1

16. Fanou EM, Coutinho JM, Shannon P, et al. Critical Illness-Associated Cerebral Microbleeds. *Stroke*. 2017;48(4):1085-1087. doi:10.1161/STROKEAHA.116.016289

17. Fitsiori A, Pugin D, Thieffry C, Lalive P, Vargas MI. COVID-19 is Associated with an Unusual Pattern of Brain Microbleeds in Critically Ill Patients. *J Neuroimaging Off J Am Soc Neuroimaging*. 2020;30(5):593-597. doi:10.1111/jon.12755

18. Gedansky A, Huang M, Hassett CE, et al. Cerebral microbleeds in acute respiratory distress syndrome. *J Stroke Cerebrovasc Dis*. 2023;32(10):107332. doi:10.1016/j.jstrokecerebrovasdis.2023.107332

19. Gijs J, Lambert J, Meyfroidt G, Demeestere J. Cerebral microbleeds and intracerebral hemorrhage associated with veno-venous extracorporeal membrane oxygenation. *Acta Neurol Belg*. 2018;118(3):513-515. doi:10.1007/s13760-018-0975-z

20. Gupta NA, Lien C, Iv M. Critical illness-associated cerebral microbleeds in severe COVID-19 infection. *Clin Imaging*. 2020;68:239-241. doi:10.1016/j.clinimag.2020.08.029

21. Hall JP, Minhas P, Kontzialis M, Jhaveri MD. Teaching NeuroImages: Distinct brain microhemorrhage pattern in critical illness associated with respiratory failure. *Neurology*. 2018;90(22):e2011. doi:10.1212/WNL.0000000000005609

22. Haroon KH, Patro SN, Hussain S, Zafar A, Muhammad A. Multiple Microbleeds: A Serious Neurological Manifestation in a Critically Ill COVID-19 Patient. *Case Rep Neurol*. 2020;12(3):373-377. doi:10.1159/000512322

23. Ippolito A, Urban H, Ghoroghi K, et al. Prevalence of acute neurological complications and pathological neuroimaging findings in critically ill COVID-19 patients with and without VV-ECMO treatment. *Sci Rep*. 2022;12(1):17423. doi:10.1038/s41598-022-21475-y

24. Jegatheeswaran V, Chan MWK, Chakrabarti S, Fawcett A, Chen YA. Neuroimaging Findings of Hospitalized Covid-19 Patients: A Canadian Retrospective Observational Study. *Can Assoc Radiol J*. 2022;73(1):179-186. doi:10.1177/08465371211002815

25. Keller E, Brandi G, Winklhofer S, et al. Large and Small Cerebral Vessel Involvement in Severe COVID-19: Detailed Clinical Workup of a Case Series. *Stroke*. 2020;51(12):3719-3722. doi:10.1161/STROKEAHA.120.031224

26. Klinkhammer S, Horn J, Duits AA, et al. Neurological and (neuro)psychological sequelae in intensive care and general ward COVID-19 survivors. *Eur J Neurol*. 2023;30(7):1880-1890. doi:10.1111/ene.15812

27. Kurki SN, Kantonen J, Kaivola K, et al. APOE epsilon4 associates with increased risk of severe COVID-19, cerebral microhaemorrhages and post-COVID mental fatigue: a Finnish biobank, autopsy and clinical study. *Acta Neuropathol Commun*. 2021;9(1):199. doi:10.1186/s40478-021-01302-7

28. Guennec LL, Bertrand A, Laurent C, et al. Diffuse cerebral microbleeds after extracorporeal membrane oxygenation support. *Am J Respir Crit Care Med*. 2015;191(5):594-596. doi:10.1164/rccm.201411-2118LE

29. Lersy F, Willaume T, Brisset JC, et al. Critical illness-associated cerebral microbleeds for patients with severe COVID-19: etiologic hypotheses. *J Neurol*. 2021;268(8):2676-2684. doi:10.1007/s00415-020-10313-8

30. Lersy F, Bund C, Anheim M, et al. Evolution of Neuroimaging Findings in Severe COVID-19 Patients with Initial Neurological Impairment: An Observational Study. *Viruses*. 2022;14(5):949. doi:10.3390/v14050949

31. Lin E, Lantos JE, Strauss SB, et al. Brain Imaging of Patients with COVID-19: Findings at an Academic Institution during the Height of the Outbreak in New York City. *AJNR Am J Neuroradiol*. Published online 2020. doi:10.3174/ajnr.A6793

32. Manelli F, Cotelli MS, Lodoli G, Bonetti S, Turla M. Cerebral microbleeds after COVID-19 infection: An Italian case report. *Ital J Med*. 2021;15(3):191-192. doi:10.4081/itjm.2021.1466

33. Myers S, Bhalla M, Jolly R, Jain S. Anisocoria in an intubated patient with COVID-19. *BMJ Case Rep*. 2021;14(7):e240003. doi:10.1136/bcr-2020-240003

34. Neligan A, Rajakulendran S, Nortley R, Manji H. Extensive cerebral microhemorrhages caused by acute disseminated intravascular coagulation secondary to sepsis. *JAMA Neurol*. 2014;71(4):510-511. doi:10.1001/jamaneurol.2013.223

35. Ong BA, Geocadin R, Choi CW, Whitman G, Cho SM. Brain magnetic resonance imaging in adult survivors of extracorporeal membrane oxygenation. *Perfusion*. 2021;36(8):814-824. doi:10.1177/0267659120968026

36. Parasram M, Loiseau SY. Cerebral microhemorrhages in severe COVID-19 pneumonia. *Surg Neurol Int*. 2022;13:380. doi:10.25259/SNI_605_2022

37. Radmanesh A, Derman A, Lui YW, et al. COVID-19 -associated Diffuse Leukoencephalopathy and Microhemorrhages. *Radiology*. Published online 2020:202040. doi:10.1148/radiol.2020202040

38. Riech S, Hellen P, Moerer O, et al. Microhemorrhages in the corpus callosum after treatment with extracorporeal membrane oxygenation. *Crit Care*. 2015;19(SUPPL. 1):S97. doi:10.1186/cc14357

39. Salam S, Rauf Y, Gulati DK. Dic presenting with diffuse cerebral microhemorrhage in a patient with sickle cell disease and thalassemia. *Interv Neurol*. 2017;6(Supplement 1):18. doi:10.1159/000477925

40. Shafaat O, Sotoudeh H, Zandifar A, et al. Critical illness-associated cerebral microbleed in a patient with sickle cell disease: A case report and review of the literature. *Clin Imaging*. 2020;68:184-187. doi:10.1016/j.clinimag.2020.07.014

41. Shah J, Armstrong MJ. Extracorporeal membrane oxygenation: uncommon cause of corpus callosal microhemorrhage. *Neurology*. 2015;84(6):630. doi:10.1212/WNL.0000000000001227

42. Shindo A, Suzuki K, Iwashita Y, Tomimoto H. Sepsis-Associated Encephalopathy with Multiple Microbleeds in Cerebral White Matter. *Am J Med*. 2018;131(7):e297-e298. doi:10.1016/j.amjmed.2018.02.012

43. Shoskes A, Huang M, Gedansky A, et al. MRI of Cerebrovascular Injury Associated With COVID-19 and Non-COVID-19 Acute Respiratory Distress Syndrome: A Matched Case-Control Study. *Crit Care Med*. 2022;50(11):1638-1643. doi:10.1097/CCM.0000000000005658

44. Sierra-Gomez A, Villanueva Rincon JM, Lopez-Mesonero L, et al. CEREBRAL MICROBLEED PHENOMENON IN CRITICALLY ILL PATIENT. *Eur Stroke J*. 2022;7(1 SUPPL):513. doi:10.1177/23969873221087559

45. Thurnher MM, Boban J, Roggla M, Staudinger T. Distinct pattern of microsusceptibility changes on brain magnetic resonance imaging (MRI) in critically ill patients on mechanical ventilation/oxygenation. *Neuroradiology*. 2021;63(10):1651-1658. doi:10.1007/s00234-021-02663-5

46. Toeback J, Depoortere SD, Vermassen J, Vereecke EL, Van Driessche V, Hemelsoet DM. Microbleed patterns in critical illness and COVID-19. *Clin Neurol Neurosurg*. 2021;203:106594. doi:10.1016/j.clineuro.2021.106594

47. Topiwala K, Hussein H, Masood K, et al. Patterns and Predictors of Extra-Corporeal Membrane Oxygenation Related Cerebral Microbleeds. *J Stroke Cerebrovasc Dis*. 2022;31(1):106170. doi:10.1016/j.jstrokecerebrovasdis.2021.106170

48. Trevino-Peinado C, Zubieta JL, Fernandez MM. Subcortical Microbleeds in Disseminated Intravascular Coagulation Mimicking Amyloid Angiopathy. *J Neuroimaging*. 2015;25(4):660-661. doi:10.1111/jon.12208

49. Vattoth S, Abdelhady M, Alsoub H, Own A, Elsotouhy A. Critical illness-associated cerebral microbleeds in COVID-19. *Neuroradiol J*. 2020;33(5):374-376. doi:10.1177/1971400920939229
